# Supplementary material for: Global analyses of genomic and epigenomic influences on gene expression reveal Serpina3n as a major regulator of cardiac gene expression in response to catecholamine challenge during heart failure
Source: Epigenetics. 2026 Mar 19;21(1):2643094. doi: 10.1080/15592294.2026.2643094 (PMC13003858; doi:10.1080/15592294.2026.2643094)
Supplement: Supplemental Data.docx [file KEPI_A_2643094_SM6657.docx]

**Supplemental Data**


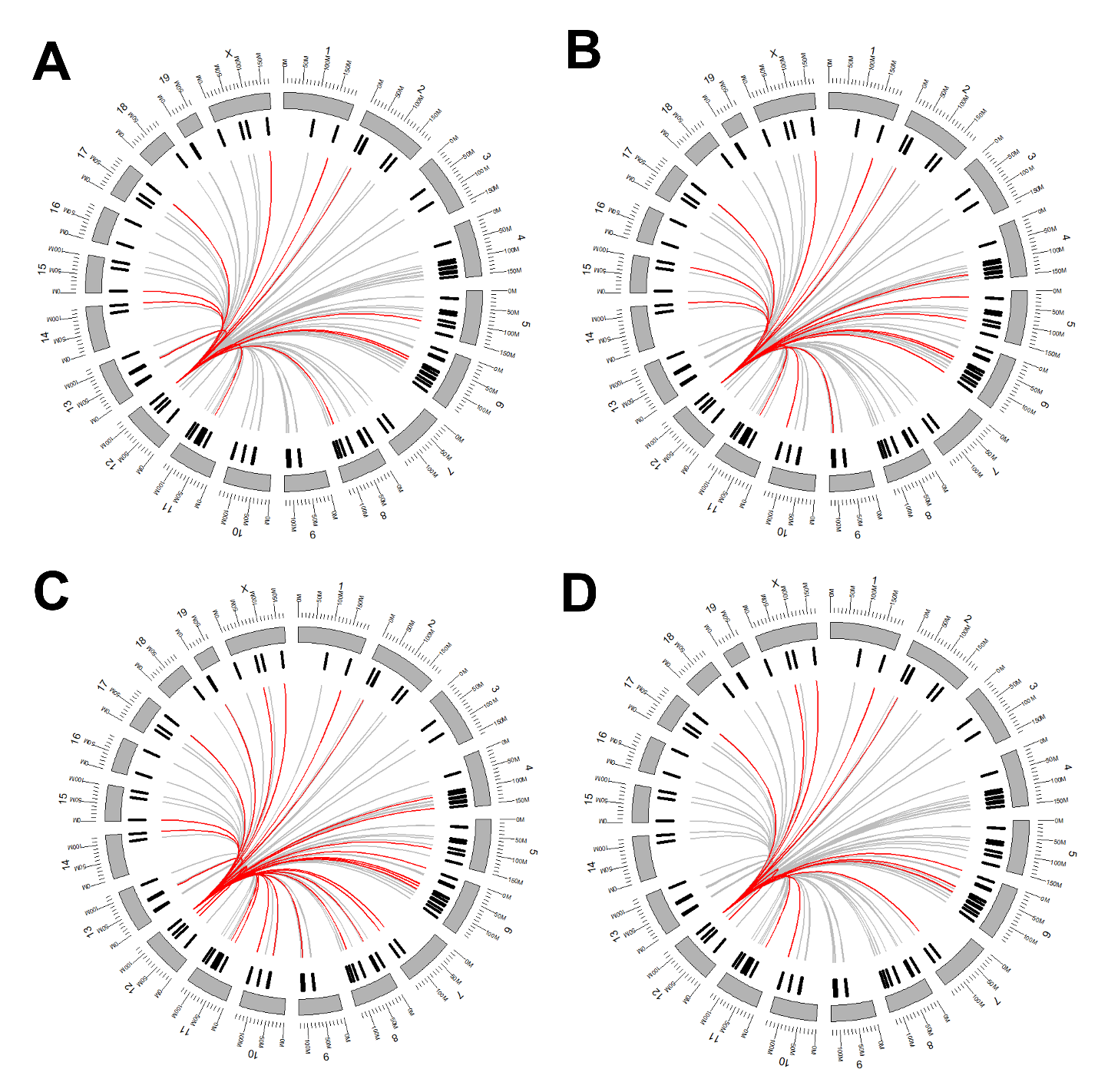


**Figure S1: Circle Ideograms for Selected GO Terms.** Ideograms generated for specific GO terms from the genome-wide significant genes linked to the *Serpina3n* locus on Chr12. A) Fatty Acid Oxidation B) Cardiomyopathy C) Mitochondrial Regulation D) ATP Synthesis


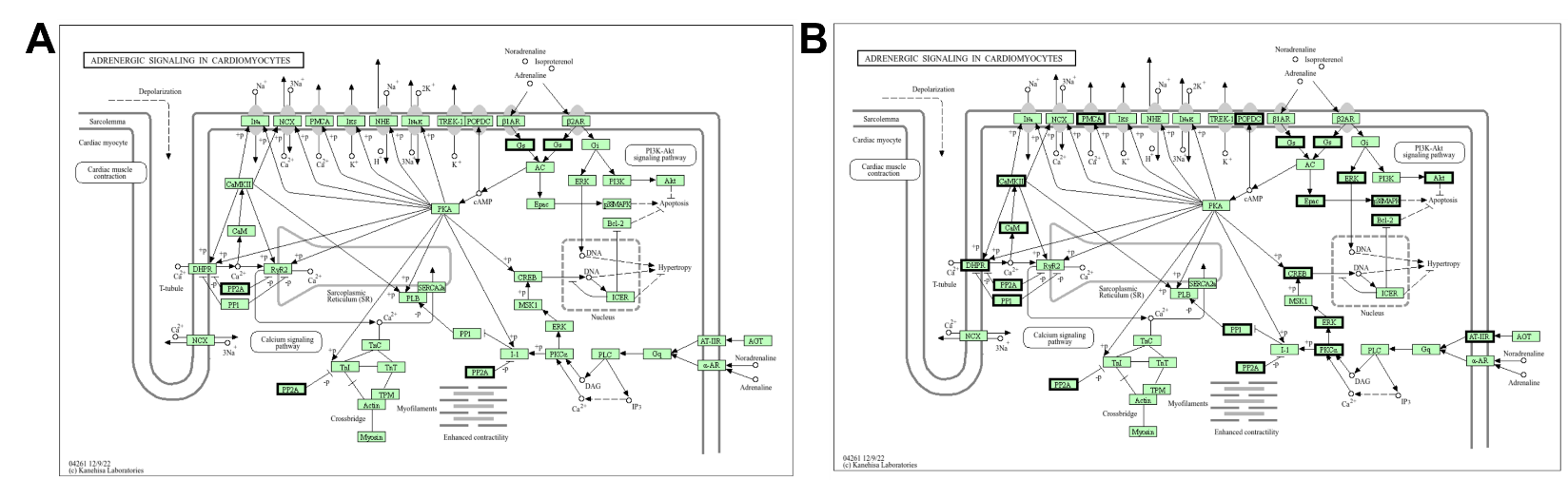


**Figure S2: KEGG Enrichments of the Adrenergic Signaling in Cardiomyocytes pathway. A)** Enrichment at the original 4.2E-5 threshold **B)** Enrichment at the relaxed 4.2E-3 threshold. Entries with bolded boxes are present in the *Serpina3n* hotspot

**Table S1:** **List of strains used in the study**

**Table S2: All sequences used in the study (primers + siRNAs)**

**Table S3: All significant peaks identified in the study**

**Table S4: Overlapping peaks with GWAS/EWAS or hypertrophic gene module results**

**Table S5: Enrichments of Genes associated with the *Serpina3n* hotspot as measured by GeneAnalytics**

**Table S6: All Values and Significances for Fig 4B**

**Table S7: All Values and Significances for Fig 4C-M**

**Table S8: GO Ontology Enrichments for all hotspots as measured by ClusterProfiler**

**Table S9: Hotspots which are present in more than one ‘Omics layer**

**Table S10: Correlations between Serpina3n and members of the Adrenergic Signaling in Cardiomyocytes KEGG Pathway**
